# Retraction Note to: A U5 repressor of reverse transcription is required for optimal HIV-1 infectivity and replication

**DOI:** 10.1186/s12977-016-0314-5

**Published:** 2016-11-10

**Authors:** Luke Meredith, Céline Ducloux, Catherine Isel, Roland Marquet, David Harrich

**Affiliations:** 1 Griffith Medical Research College, A Joint Program of Griffith University and the Queensland Institute of Medical Research, QIMR, Herston, QLD 4006 Australia; 2 Queensland Institute of Medical Research, Royal Brisbane Hospital Post Office, Brisbane, QLD 4029 Australia; 3 Architecture et Réactivité de l’ARN, Université de Strasbourg, CNRS, IBMC, 15 rue René Descartes, 67084 Strasbourg Cedex, France

## Retraction Note to: Retrovirology 2009, **6**(Suppl 2):O14 doi:10.1186/1742-4690-6-S2-O14

The authors are retracting this abstract [1]. The preliminary data reported were presented at the “Frontiers of Retrovirology: Complex Retroviruses, Retroelements and Their Hosts”, in September 2009. Subsequently, the authors have been unable to reproduce key cell culture data and have discontinued this project. All of the authors agree with this retraction.
